# Preclinical translational screening of palladium(II)-porphyrin photosensitizers across human and Oncopig bladder cancer cell lines

**DOI:** 10.3389/fonc.2026.1882402

**Published:** 2026-07-09

**Authors:** Valentina G. Ferreira, Maria Eduarda Ehlert, Bruna S. Pacheco, Fernanda S. S. Souza, Isadora Tisoco, Lucas Damé Simões, Bernardo A. Iglesias, Claudia Ó. Pessoa, Fabiana K. Seixas, Maria Lucia Z. Dagli, Laurie A. Rund, Kyle M. Schachtschneider, Lawrence B. Schook, Tiago Veiras Collares

**Affiliations:** 1 Molecular and Cellular Oncology Research Group, Cancer Biotechnology Laboratory, Technological Development Center, Federal University of Pelotas, Pelotas, Brazil; 2 INCT T-Bio2: Translational Biodiscovery and Biomodels, Pelotas, Rio Grande do Sul, Brazil; 3 Department of Chemistry, Federal University of Santa Maria, Santa Maria, Brazil; 4 Department of Chemistry, Federal University of Santa Catarina, Florianópolis, Brazil; 5 Department of Physiology and Pharmacology, Faculty of Medicine, Federal University of Ceará, Fortaleza, Brazil; 6 Department of Pathology, School of Veterinary Medicine and Animal Science, University of São Paulo, São Paulo, SP, Brazil; 7 Department of Animal Sciences, University of Illinois, Urbana, IL, United States; 8 Sus Clinicals Inc., Chicago, IL, United States

**Keywords:** bladder cancer, Oncopig cancer model, palladium(II)-porphyrins, photodynamic therapy, preclinical screening, translational oncology

## Abstract

**Background:**

Bladder cancer remains a highly recurrent malignancy with limited long-term response to conventional therapies. The development of new treatments is also hindered by the lack of preclinical models that accurately reproduce human disease. In this context, Oncopig-derived models provide a translationally relevant platform for evaluating candidate therapies before progression to *in vivo* studies.

**Methods:**

Here, we performed a preclinical *in vitro* screening of three palladium(II)-porphyrins, 3-Pd(PPh_3_), 3-Pd(dppf), and 3-Pd(PEPSI), in human (T24, RT4, 5637) and Oncopig-derived bladder cancer cell models. Cytotoxicity was assessed under light and dark conditions using MTT and LIVE/DEAD assays, while intracellular ROS generation was quantified via DCFH-DA fluorescence. Gene expression profiling (qRT-PCR) targeted oxidative stress and apoptosis markers (SOD, CAT, GPx, BAX, BCL2, CASP3/8/9), and nuclear morphology was analyzed using DAPI staining.

**Results:**

All three compounds exhibited light-dependent cytotoxicity with minimal dark toxicity. While 3-Pd(PPh_3_) and 3-Pd(dppf) demonstrated potent photodynamic activity with nanomolar IC_50_ values, 3-Pd(PEPSI) showed consistently higher IC_50_ values across the tested bladder cancer cell lines, indicating lower photodynamic potency under the evaluated conditions. Photoactivation triggered significant ROS production and upregulation of CAT and CASP3, alongside increased BAX/BCL2 ratios, supporting a ROS-mediated apoptotic mechanism. Notably, Oncopig-derived cells exhibited molecular and phenotypic responses closely matching those of human bladder cancer cell lines.

**Conclusion:**

These findings support the translational potential of Pd(II)-porphyrins for bladder cancer PDT and position Oncopig-derived *in vitro* models as a responsible preclinical screening layer to prioritize photosensitizers before future large-animal validation of localized and image-guided PDT strategies.

## Graphical Abstract

**Figure:**
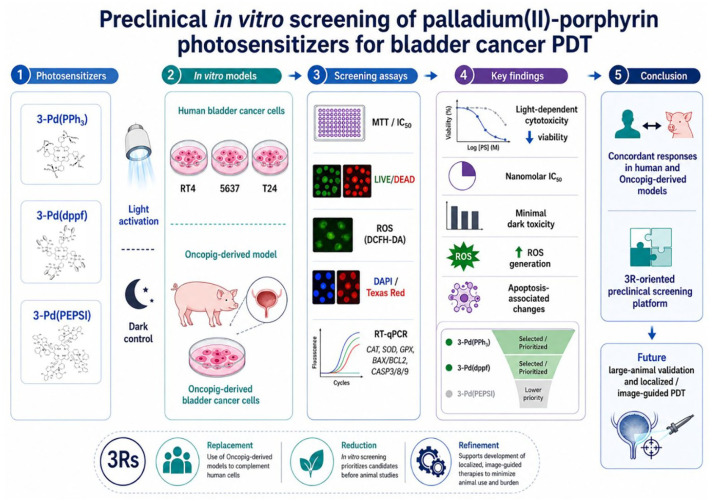

## 1 Introduction

Bladder cancer (BC) is one of the most prevalent malignancies of the urinary tract and ranks among the ten most common cancers worldwide, with over 570,000 new cases and 210,000 deaths annually (1). Despite advances in diagnosis and therapy, the disease still presents high recurrence and progression rates, particularly in muscle-invasive forms, which account for increased mortality and limited therapeutic success (2). Conventional treatments, such as transurethral resection, chemotherapy, and intravesical immunotherapy with Bacillus Calmette-Guérin (BCG), often cause severe side effects and fail to ensure long-term control of the disease (3). Therefore, the development of novel and less invasive therapeutic approaches remains an urgent need.

However, the successful translation of emerging therapies for bladder cancer remains strongly dependent on the availability of preclinical models that accurately recapitulate the complexity of the human disease. Traditional murine models, while widely used because of their low cost, genetic tractability, and rapid tumor development, have significant limitations in replicating human anatomy, urinary physiology, and immune responses (4). In particular, the reduced bladder volume and anatomical dimensions of mice restrict the realistic assessment of drug delivery systems, imaging-guided interventions, and light-based therapies such as photodynamic therapy (5, 6). These limitations highlight the need for translational models capable of bridging the gap between conventional *in vitro* assays, rodent studies, and clinically relevant preclinical development.

Among large-animal models, swine have gained increasing attention in translational oncology because of their remarkable similarity to humans in terms of body size, organ architecture, urinary tract anatomy, physiology, metabolism, immune system function, and drug response, further increasing their translational relevance for preclinical cancer research (7, 8). In this scenario, the Oncopig Cancer Model (OCM), a transgenic pig harboring inducible KRAS^G12D^ and TP53^R167H^ mutations, has emerged as a powerful platform for cancer research (8–10). Notably, Oncopig-derived bladder cancer cell lines reproduce key molecular, genetic, and histopathological features of human bladder cancer, supporting their use as relevant tools for preclinical therapeutic evaluation (11).

Importantly, Oncopig-derived *in vitro* models may also contribute to a more responsible preclinical development pipeline aligned with the principles of replacement, reduction, and refinement (12, 13). Rather than advancing multiple candidate photosensitizers directly into animal studies, these cell-based models allow an initial mechanistic screening step in which phototoxic potency, dark toxicity, reactive oxygen species generation, apoptotic response, and molecular concordance with human bladder cancer cells can be assessed under controlled experimental conditions (14). This strategy supports replacement by using cell-based assays for early exploratory testing, reduction by prioritizing only the most promising compounds for subsequent *in vivo v*alidation, and refinement by informing concentration ranges, light exposure conditions, biological endpoints, and safety considerations before large-animal studies are performed (13).

In this context, photodynamic therapy (PDT) is a promising approach based on the interaction of a photosensitizer (PS), light, and molecular oxygen, generating reactive oxygen species (ROS) that induce tumor cell death (15). PDT is minimally invasive, repeatable, and promotes localized tumor destruction while preserving healthy tissues. Particularly in bladder cancer, PDT represents an attractive therapeutic strategy due to the accessibility of the bladder lumen, which enables localized light delivery and supports the future development of image-guided therapeutic approaches (5, 16).

However, PDT efficacy depends strongly on the physicochemical and photobiological properties of the photosensitizer used (14, 17). Among the various classes of photosensitizers, porphyrin-based compounds have attracted considerable attention due to their favorable photophysical properties, efficient ROS generation, tunable chemical structures, and potential for enhanced cellular interaction and tumor selectivity (18, 19).

In this context, the present study aimed to perform a preclinical *in vitro* screening of three meso-tetra-cationic palladium porphyrins, 3-Pd(PPh_3_), 3-Pd(dppf), and 3-Pd(PEPSI), using human and Oncopig-derived bladder cancer models. Cytotoxicity, dark toxicity, ROS generation, nuclear morphology, and oxidative stress- and apoptosis-related gene expression were analyzed to identify photosensitizers with sufficient biological activity and translational plausibility to justify progression to future large-animal validation.

## 2 Materials and methods

### 2.1 Meso-Tetra-cationic porphyrin-based photosensitizers with peripheral palladium(II) complexes

The meso-tetra-cationic porphyrin-based photosensitizers bearing peripheral palladium(II) complexes were synthesized and characterized by our research group. The structural, photophysical, and photostability properties of these compounds were previously reported (20). All porphyrins were dissolved in dimethyl sulfoxide (DMSO) (Neon, Brazil; Cat. No. 03014) to prepare stock solutions at 1.0 mg/mL and intermediate stock solutions at 150 µM. These solutions were subsequently diluted in the appropriate culture medium to obtain the desired working concentrations.

### 2.2 Cell culture

The human bladder cancer cell lines T24, RT4, and 5637 were obtained from the Rio de Janeiro Cell Bank (PABCAM, Federal University of Rio de Janeiro, RJ, Brazil). The Oncopig-derived bladder cancer cell line was kindly provided by the University of Illinois (Chicago, USA) and was originally established from bladder tissue collected from Oncopigs as previously described (31). The original derivation of this cell line was performed under an approved University of Illinois Institutional Animal Care and Use Committee (IACUC) protocol (31). No animals were generated or used specifically for the present study, which was conducted exclusively using previously established cell lines. RT4 and T24 cells were cultured in Dulbecco’s Modified Eagle Medium (DMEM) (Vitrocell Embriolife, Campinas, Brazil, Cat. No. D2861), while 5637 cells were maintained in Roswell Park Memorial Institute (RPMI) 1640 medium (Vitrocell Embriolife, Campinas, Brazil, Cat. No. R0009). Oncopig-derived cells were cultured in high-glucose DMEM (Vitrocell Embriolife, Campinas, Brazil, Cat. No. D2828). All culture media were supplemented with 10% fetal bovine serum (FBS) (Vitrocell Embriolife, Campinas, Brazil, Cat. No. S0011). Cells were maintained under standard culture conditions at 37 °C, 95% relative humidity, and 5.0% CO_2_ atmosphere.

### 2.3 Experimental groups and photodynamic assay

The experimental procedure was conducted following the methodology previously established by our research group (21). The study was divided into two experimental conditions: a light-exposed group and a dark, non-irradiated group. After 24 h of cell seeding and adherence, each group was treated with seven concentrations (200, 100, 50, 28, 14, 7, and 3.5 nM) of the three meso-tetra-cationic palladium(II) porphyrins. The selected concentration range was based on preliminary screening experiments and the previously reported biological activity of structurally related palladium- and platinum-containing porphyrins developed by our research group (20, 21). This range was designed to encompass concentrations below and above the expected IC_50_ values, allowing reliable nonlinear dose-response curve fitting while maintaining the final DMSO concentration below 0.5% Since these photosensitizers are activated upon light exposure, the light-treated group underwent a photodynamic irradiation session. During this procedure, the porphyrins were illuminated using a broad-spectrum white LED light source (400–800 nm) emitted by a 100 W LED lamp system, delivering an irradiance of 50 mW/cm² for 30 min at a distance of 15 cm from the culture plate, corresponding to a total light fluence of 90 J/cm². Following irradiation, the plates were returned to the incubator, and subsequent analyses were performed 24 h later. The dark group was maintained under the same experimental conditions but protected from light exposure.

### 2.4 MTT colorimetric assay

The MTT colorimetric assay was performed to evaluate cell viability based on the conversion of the yellow tetrazolium salt 3-[4,5-dimethylthiazol-2-yl]-2,5-diphenyltetrazolium bromide (MTT) (Sigma-Aldrich, Cat. No. CT475989) into purple-blue formazan crystals by metabolically active cells. Cells were seeded in 96-well plates at densities of 2.0 × 10^4^ cells/well for RT4, T24, and 5637 cell lines, and 1.0 × 10^4^ cells/well for the Oncopig-derived cell line. Treatments and photodynamic conditions were performed as described in Section 2.3. Blank wells contained culture medium only, while vehicle controls contained culture medium supplemented with DMSO, maintaining the final DMSO concentration below 0.5% per well. Cell viability was assessed 24 h after photodynamic activation, corresponding to 48 h after seeding. MTT solution (5.0 mg/mL) was then added to each well, followed by 3 h of incubation. Absorbance was measured at 492 nm using a microplate reader (Thermo Plate TP-Reader). All experiments were performed in three independent biological replicates, with each experimental condition analyzed in technical triplicate. The percentage of cell growth inhibition was calculated using the following equation:


$$\text{\% inhibition}=\left(1‐{\text{Abs}}_{492}\text{ of treated }{\text{cells/Abs}}_{492}\text{ of control cells}\right)\;\times 100$$

### 2.5 LIVE/DEAD cell viability assay

The LIVE/DEAD cell viability assay (Invitrogen™, Carlsbad, USA, Cat. No. L3224) was performed to discriminate between viable and non-viable cells based on cell membrane integrity. Cells were seeded in 96-well plates following the same conditions described for the MTT assay and treated with 3-Pd(PPh_3_) and 3-Pd(dppf) at concentrations corresponding to the IC_50_ values determined for each cell line. The LIVE/DEAD assay was carried out 24 h after photodynamic activation of the compounds. In this assay, viable cells convert non-fluorescent calcein-AM into fluorescent green calcein, detected at 488 nm. In contrast, dead cells are stained with ethidium homodimer-1 (EthD-1), which penetrates damaged membranes and binds to DNA, emitting red fluorescence at 546 nm. The LIVE/DEAD working solution was prepared according to the manufacturer’s instructions by mixing 2.0 μL of calcein-AM and 0.5 μL of EthD-1 in 1.0 mL of PBS. Cell images were acquired using a Leica TCS SP8 laser scanning confocal microscope, and image analysis was performed using ImageJ software. Three distinct fields of view were analyzed per sample, and red-stained dead cells were quantified to calculate the cell death rate. The assay was performed in three independent biological replicates, with each condition analyzed in technical triplicate.

### 2.6 Fluorescent staining for nuclear and cytoplasmic/cytoskeletal morphology

Changes in nuclear and cytoplasmic/cytoskeletal morphology indicative of cell death were evaluated by dual staining with DAPI (4′,6-diamidino-2-phenylindole) (Invitrogen™, Cat. No. D1306) and Texas Red™-X Phalloidin (Invitrogen™, Cat. No. T7471). DAPI selectively binds to double-stranded DNA, forming a fluorescent complex that enables visualization of nuclear condensation and fragmentation, which are characteristic of apoptotic nuclei. Texas Red™-X Phalloidin staining was used to visualize F-actin organization and evaluate cytoplasmic/cytoskeletal architecture and overall cellular morphology. Cells were seeded in 96-well plates and treated with the IC_50_ concentrations of 3-Pd(PPh_3_) and 3-Pd(dppf) for 24 h. Subsequently, cells were exposed to photodynamic activation and incubated for an additional 24 h. After treatment, cells were washed three times with PBS 1×, fixed with paraformaldehyde and Triton X-100, and stained following the manufacturer’s instructions. Fluorescent images were acquired using a Leica TCS SP8 confocal microscope. Nuclear morphology and cytoplasmic/cytoskeletal organization were analyzed to identify treatment-associated morphological changes. DAPI fluorescence intensity was used as a complementary imaging parameter to assess treatment-associated nuclear alterations and was not interpreted as a quantitative stand-alone measure of apoptosis. Three distinct fields of view were analyzed per sample. Experiments were conducted in three independent biological replicates, with each condition analyzed in technical triplicate.

### 2.7 Evaluation of intracellular ROS generation

Oncopig-derived and human bladder cancer cell lines were seeded in 96-well plates under the same conditions described for the MTT assay. Cells were treated with 3-Pd(PPh_3_) and 3-Pd(dppf) at concentrations corresponding to the IC_50_ values previously determined for each cell line. Cultures were maintained at 37 °C in a humidified incubator with 5.0% CO_2_ for 24 h. The DCFH-DA (2′,7′-dichlorofluorescein diacetate) (Invitrogen™, Cat. No. D399) assay was performed 24 h after photodynamic activation of the photosensitizers. To assess intracellular ROS generation, cells were incubated with 10 μM (DCFH-DA) at 37 °C for 30 min in the dark. The excess probe was removed by washing the cells twice with PBS 1× to eliminate unbound dye. Fluorescence images were acquired using a Leica TCS SP8 laser scanning confocal microscope, and DCF fluorescence intensity was used as an indicator of intracellular ROS production. Three distinct fields of view were analyzed per sample. DCFH-DA fluorescence intensity was normalized to the number of cells in each field to account for differences in cell density between conditions. Experiments were conducted in three independent biological replicates, with each condition analyzed in technical triplicate.

### 2.8 Gene expression analysis

For gene expression studies, all cell lines were seeded in 6-well plates at a density of 5 × 10^5^ cells per well. Cells were treated with the porphyrins at their respective IC_50_ concentrations, and photodynamic activation was performed 24 h post-treatment. Samples were collected 24 h after photodynamic activation. Total RNA was extracted using TRIzol reagent (Invitrogen™, Carlsbad, USA, Cat. No.15596026) according to the manufacturer’s instructions. RNA concentration and purity were assessed using a NanoVue Plus™ spectrophotometer (GE Healthcare). cDNA synthesis was performed using the High-Capacity cDNA Reverse Transcription Kit (Applied Biosystems™, UK, Cat. No. 4374967). Quantitative real-time PCR (RT-qPCR) was carried out on a Stratagene Mx3005P Real-Time PCR System (Agilent Technologies, Santa Clara, CA, USA) using SYBR Green PCR Master Mix (Applied Biosystems™, UK, Cat. No. 10607705). The oxidative stress-related genes were CAT, SOD, and GPx; the apoptosis-associated genes were BAX, BCL2, CASP3, CASP8, and CASP9; and GAPDH was used as the reference gene. Primer sequences, including forward and reverse oligonucleotides used in this study, are presented in the Supplementary Information, **Supplementary Table 1**. All primers were synthesized by Exxtend (São Paulo, Brazil). RNA samples were obtained from three independent biological replicates, and all RT-qPCR reactions were performed in a technical triplicate. Relative gene expression levels were calculated using the 2^−ΔΔCt method, as described by Livak and Schmittgen (22).

### 2.9 Statistical analysis

All statistical analyses were performed using GraphPad Prism 8 (GraphPad Software Inc., San Diego, CA, USA), with the significance level set at p < 0.05. For the MTT assay, data were analyzed using one-way ANOVA followed by Tukey’s *post hoc* test for multiple comparisons. IC_50_ values were calculated by nonlinear regression using variable-slope sigmoidal dose-response curves and are reported together with their 95% confidence intervals (95% CI) (**Table 1**). LIVE/DEAD quantification, DAPI/Texas Red fluorescence analysis, and intracellular ROS generation assessed by DCFH-DA were analyzed using two-way ANOVA followed by Tukey’s *post hoc* test and are presented as mean ± SD. RT-qPCR data were analyzed using two-way ANOVA followed by Tukey’s *post hoc* test and are presented as mean ± standard error of the mean (SEM). Statistical significance is indicated as *p < 0.05, **p < 0.01, and ***p < 0.001.

**Table 1:** IC_50_ values of the compounds 3-Pd(PPh_3_), 3-Pd(dppf), and 3-Pd(PEPSI) after 24 hours of light exposure in the Oncopig, RT4, 5637 and T24, RT4 cell lines.

Cell line	Porphyrin	IC_50_ (nM)	95% CI (nM)
*Oncopig Bladder Cancer Cell Line*	3-Pd(PPh_3_)	16,49	12,41 - 21,90
	3-Pd(dppf)	15,88	13,49 - 18,69
	3-Pd(PEPSI)	73,44	67,36 - 80,28
RT4	3-Pd(PPh_3_)	16,68	15,03 - 18,51
	3-Pd(dppf)	14,34	12,95 - 15,87
	3-Pd(PEPSI)	57,62	52,12 - 63,71
5637	3-Pd(PPh_3_)	8,31	7,047 - 9,791
	3-Pd(dppf)	15,79	14,09 - 17,69
	3-Pd(PEPSI)	36,48	32,68 - 40,73
T24	3-Pd(PPh_3_)	54,87	48,44 - 62,16
	3-Pd(dppf)	40,47	35,90 - 45,61
	3-Pd(PEPSI)	80,55	68,59 - 94,18

Values are presented together with their corresponding 95% confidence intervals (95% CI).

## 3 Results

**Figure 1:**
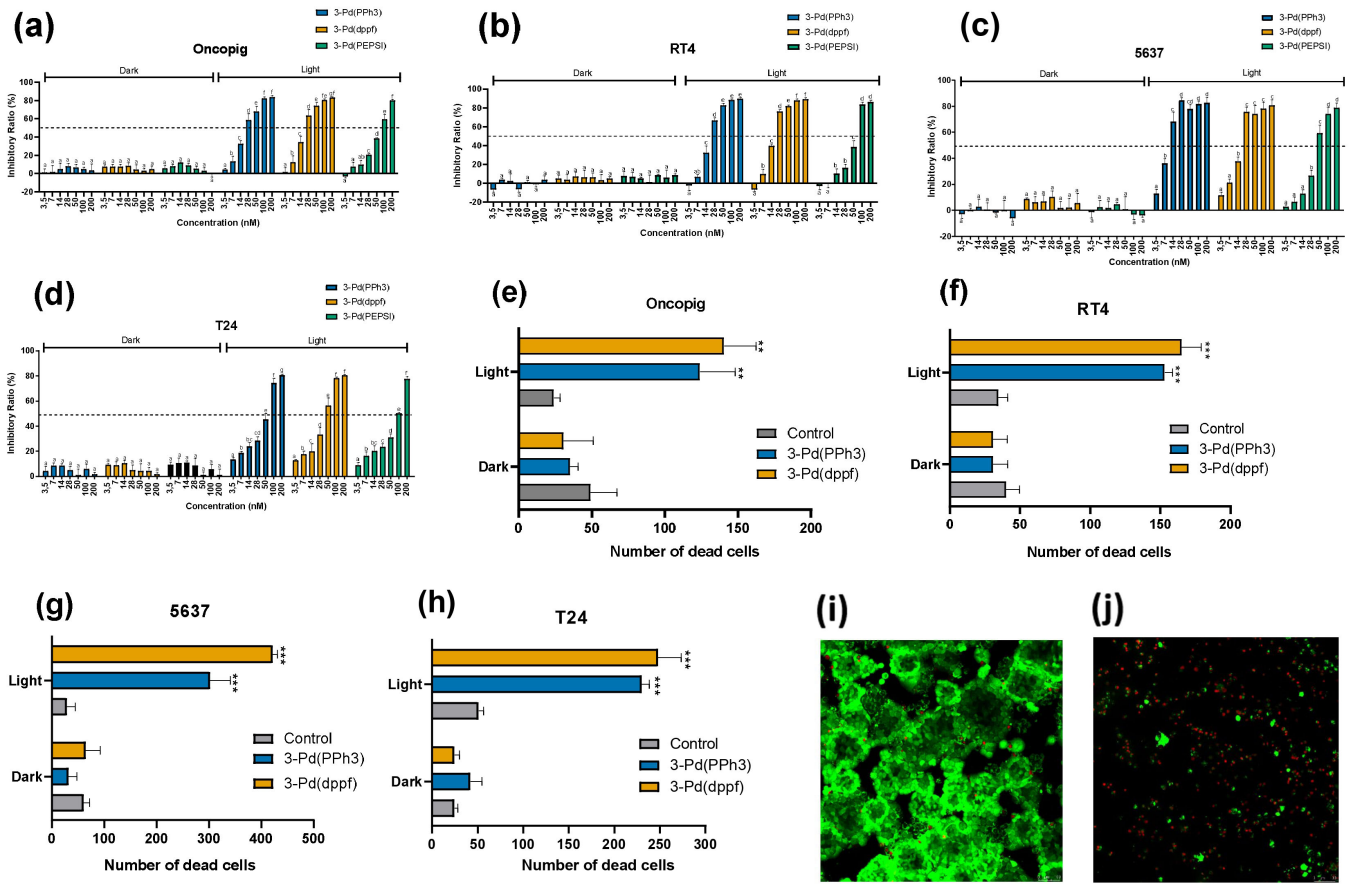
Light-dependent cytotoxicity of palladium(II)-porphyrin photosensitizers in human and Oncopig-derived bladder cancer models. Inhibitory effects of 3-Pd(PPh_3_), 3-Pd(dppf), and 3-Pd(PEPSI) were evaluated by MTT assay under dark and light conditions in **(A)** Oncopig-derived bladder cancer cells, **(B)** RT4 cells, **(C)** 5637 cells, and **(D)** T24 cells. Different lowercase letters above the bars indicate statistically significant differences among concentrations for the same compound. Statistical analysis was performed using one-way ANOVA followed by Tukey’s *post hoc* test (p < 0.05). Quantification of dead cells by LIVE/DEAD assay after treatment with 3-Pd(PPh_3_) and 3-Pd(dppf) under dark and light conditions in **(E)** Oncopig-derived bladder cancer cells, **(F)** RT4 cells, **(G)** 5637 cells, and **(H)** T24 cells. Data are presented as mean ± SD from three independent biological replicate. Statistical significance was determined by two-way ANOVA followed by Tukey’s *post hoc* test. *p < 0.05; **p < 0.01; ***p < 0.001. Representative confocal microscopy images of RT4 cells treated with 3-Pd(dppf) under **(I)** dark and **(J)** light conditions are shown. Calcein-AM staining indicates viable cells, while EthD-1 staining indicates dead cells. Scale bars: 100 µm.

All three Pd(II)-porphyrins evaluated in this study, 3-Pd(PPh_3_), 3-Pd(dppf) and 3-Pd(PEPSI), exhibited phototoxic activity against human and Oncopig-derived bladder cancer cell lines. The MTT assay demonstrated a pronounced decrease in cell viability in light-irradiated groups treated with the compounds, whereas cells maintained under dark conditions retained viability levels comparable to untreated controls (**Figures 1A–D**). This clear distinction between light and dark conditions confirmed that the cytotoxic effects were dependent on photoactivation, as expected for photosensitizers used in PDT. Under light exposure, all compounds exhibited IC_50_ values in the nanomolar range, demonstrating their strong photodynamic activity (**Table 1**). Representative dose-response curves used for IC_50_ determination are provided in Supplementary Information, **Supplementary Figure 10**. No measurable IC_50_ values were detected under dark conditions, indicating minimal or absent dark toxicity. Among the tested compounds, 3-Pd(PEPSI) consistently exhibited higher IC_50_ values than 3-Pd(PPh_3_) and 3-Pd(dppf), indicating comparatively lower phototoxic efficiency. Therefore, based on this initial *in vitro* screening step, 3-Pd(PEPSI) was not selected for subsequent mechanistic analyses.

The LIVE/DEAD fluorescence assay corroborated the MTT findings by demonstrating increased cell death in light-irradiated groups treated with 3-Pd(PPh_3_) and 3-Pd(dppf) (**Figure 1E-H** and **Supplementary Figure 1**). In the Oncopig-derived bladder cancer cell line, quantitative analysis, expressed as mean ± SD, showed a significant increase in the number of dead cells after light exposure in the presence of 3-Pd(PPh_3_) or 3-Pd(dppf) compared with the light-only control and dark-treated groups (p = 0.002; p = 0.001, **Figure 1E**). No significant differences were observed among groups under dark conditions.

A similar response was observed in the RT4 cell line, in which light exposure combined with either 3-Pd(PPh_3_) or 3-Pd(dppf) significantly increased cell death compared with controls (both p < 0.001; **Figure 1F**). Representative confocal microscopy images of RT4 cells treated with 3-Pd(dppf) further illustrate the predominance of viable cells under dark conditions and increased EthD-1-positive dead cells after light activation (**Figures 1I, J**). In the 5637 and T24 cell lines, fluorescence microscopy revealed extensive EthD-1-positive staining after PDT treatment with both porphyrins (**Supplementary Figure 1**). Quantification, expressed as mean ± SD, confirmed that light-irradiated cells treated with 3-Pd(PPh_3_) or 3-Pd(dppf) displayed significantly higher numbers of dead cells than their respective dark controls and light-only groups (p < 0.001 for all comparisons; **Figures 1G, H**). These findings demonstrate that 3-Pd(PPh_3_) and 3-Pd(dppf) induce robust and strictly light-dependent cytotoxicity across human and Oncopig-derived bladder cancer models.

Because ROS are central mediators of PDT-induced cytotoxicity, the expression of oxidative stress-related genes (CAT, SOD and GPx) was assessed by RT-qPCR, with data expressed as mean ± SEM. In the Oncopig-derived bladder cancer cell line, photoactivation of both compounds modulated oxidative stress-related genes, although distinct expression patterns were observed. Treatment with 3-Pd(PPh_3_) increased CAT expression (p = 0.004), whereas 3-Pd(dppf) upregulated CAT (p = 0.0013) and GPx (p = 0.0028). No significant changes in SOD expression were detected for either compound. In RT4 cells, photoactivation of 3-Pd(PPh_3_) induced significant increases in CAT (p < 0.0001) and SOD (p = 0.0092), while 3-Pd(dppf) was associated with increased SOD expression (p < 0.0001) and reduced GPx expression (p = 0.0018), without significant modulation of CAT. No comparable transcriptional changes were observed under dark conditions (**Supplementary Figure 6** and **S7**).

In the 5637 cell line, photoactivation of both compounds induced oxidative stress-related responses, although with distinct expression profiles. Treatment with 3-Pd(PPh_3_) significantly increased CAT (p = 0.002), SOD (p < 0.0001), and GPx (p < 0.0001), whereas 3-Pd(dppf) upregulated CAT (p = 0.0077) and SOD (p = 0.0002) but not GPx (**Supplementary Figure 8**). In T24 cells, a significant oxidative stress response was observed only after photoactivation of 3-Pd(PPh_3_), with increased expression of CAT (p > 0.0001), SOD (p > 0.0001), and GPx (p = 0.0104), while 3-Pd(dppf) did not significantly affect the analyzed genes. No significant modulation was detected in dark-treated groups (**Supplementary Figure 9**).

Intracellular ROS generation was evaluated using the DCFH-DA fluorescence assay, with quantitative fluorescence data expressed as mean ± SD. Consistent with the gene expression data, DCFH-DA fluorescence microscopy revealed green fluorescence in all human and Oncopig-derived bladder cancer cell models treated with 3-Pd(PPh_3_) or 3-Pd(dppf) under light exposure, indicating increased intracellular ROS generation. In contrast, cells maintained under dark conditions exhibited only basal fluorescence levels comparable to untreated controls (**Supplementary Figures 4**, **5**). Quantitative analysis of DCFH-DA fluorescence intensity, expressed as mean ± SD, confirmed a statistically significant increase in intracellular ROS levels following photoactivation of both porphyrins. In the Oncopig-derived and RT4 cell lines, light-irradiated cells treated with 3-Pd(PPh_3_) or 3-Pd(dppf) showed marked elevation of ROS-associated fluorescence intensity (p < 0.001), whereas no significant changes were observed in dark-treated or light-only control groups (**Supplementary Figure 4**). A similar trend was observed in 5637 and T24 cells, in which light exposure in the presence of either porphyrin significantly increased intracellular ROS production (p < 0.001 for all comparisons), while dark-treated cells remained unaffected (**Supplementary Figure 5**).

To investigate whether the photodynamic effect of the palladium(II)-porphyrins was associated with apoptosis, the expression of key pro- and anti-apoptotic markers was quantified by RT-qPCR, with data expressed as mean ± SEM. The analysis included CASP3, CASP8, CASP9, and the BAX/BCL-2 expression ratio in human and Oncopig-derived bladder cancer models. In the Oncopig-derived bladder cancer cell line, photoactivation of both compounds promoted pro-apoptotic transcriptional responses, although with distinct profiles. Treatment with 3-Pd(PPh_3_) increased CASP3 (p = 0.0263), CASP8 (p = 0.0214), CASP9 (p = 0.006), and the BAX/BCL-2 ratio (p < 0.0001), whereas 3-Pd(dppf) increased CASP3 expression (p = 0.007) and the BAX/BCL-2 ratio (p = 0.0037) without affecting CASP8 or CASP9 (**Supplementary Figure 6**). In RT4 cells, photoactivation of 3-Pd(PPh_3_) induced a limited apoptotic response, characterized by increased CASP8 expression (p = 0.0251), while 3-Pd(dppf) promoted broader caspase activation, increasing CASP3 (p < 0.0001), CASP8 (p = 0.0001), and CASP9 (p < 0.0001) expression, with no significant changes in the BAX/BCL-2 ratio (**Supplementary Figure 7**).

In the 5637 cell line, photoactivation of both compounds significantly increased CASP3 expression, including 3-Pd(PPh_3_) (p = 0.0001) and 3-Pd(dppf) (p < 0.0001)., accompanied by an increased BAX/BCL-2 ratio following treatment with 3-Pd(PPh_3_) (p = 0.0049) and a more pronounced increase after photoactivation of 3-Pd(dppf) (p = 0.0010). However, distinct effects on initiator caspases were observed, with 3-Pd(PPh_3_) producing no significant modulation of CASP8 or CASP9, whereas 3-Pd(dppf) reduced CASP8 expression (p = 0.0010) and increased CASP9 expression (p < 0.0025) (**Supplementary Figure 8**). In T24 cells, apoptotic modulation was more selective. Photoactivated 3-Pd(PPh_3_) increased CASP3 (p < 0.0001) and CASP8 (p = 0.0050), while 3-Pd(dppf) induced a moderate increase in CASP3 (p = 0.0024) together with reduced CASP8 expression (p = 0.0173). Neither compound significantly affected CASP9 expression or the BAX/BCL-2 ratio in this cell line (**Supplementary Figure 9**). No significant transcriptional changes were detected under dark conditions, confirming the light-dependent pro-apoptotic activity of both compounds across all cell lines.

Fluorescence microscopy using DAPI and Texas Red staining provided morphological evidence consistent with the RT-qPCR findings. DAPI staining revealed increased nuclear condensation and fragmentation in porphyrin-treated cells exposed to light, while Texas Red staining highlighted cytoplasmic and cytoskeletal disorganization consistent with loss of cellular integrity (**Supplementary Figures 2**, **3**). Quantitative analysis of DAPI fluorescence intensity per cell, expressed as mean ± SD, demonstrated a significant increase following light irradiation in cells treated with either 3-Pd(PPh_3_) or 3-Pd(dppf), supporting the presence of apoptosis-associated nuclear alterations.

In the Oncopig-derived cell line, DAPI fluorescence intensity was significantly elevated in light-irradiated cells treated with 3-Pd(PPh_3_) (p = 0.04) and 3-Pd(dppf) (p = 0.001), whereas no significant changes were detected under dark conditions or in light-only controls (**Supplementary Figure 2**). In RT4 cells, both 3-Pd(PPh_3_) and 3-Pd(dppf) significantly increased DAPI fluorescence after light exposure (p < 0.001), with no comparable changes under dark conditions (**Supplementary Figure 2**). Similarly, 5637 and T24 cells showed significantly increased DAPI fluorescence intensity after light-activated treatment with both porphyrins (p < 0.001), confirming consistent nuclear alterations across human bladder cancer cell models (**Supplementary Figure 3**).

**Figure 2:**
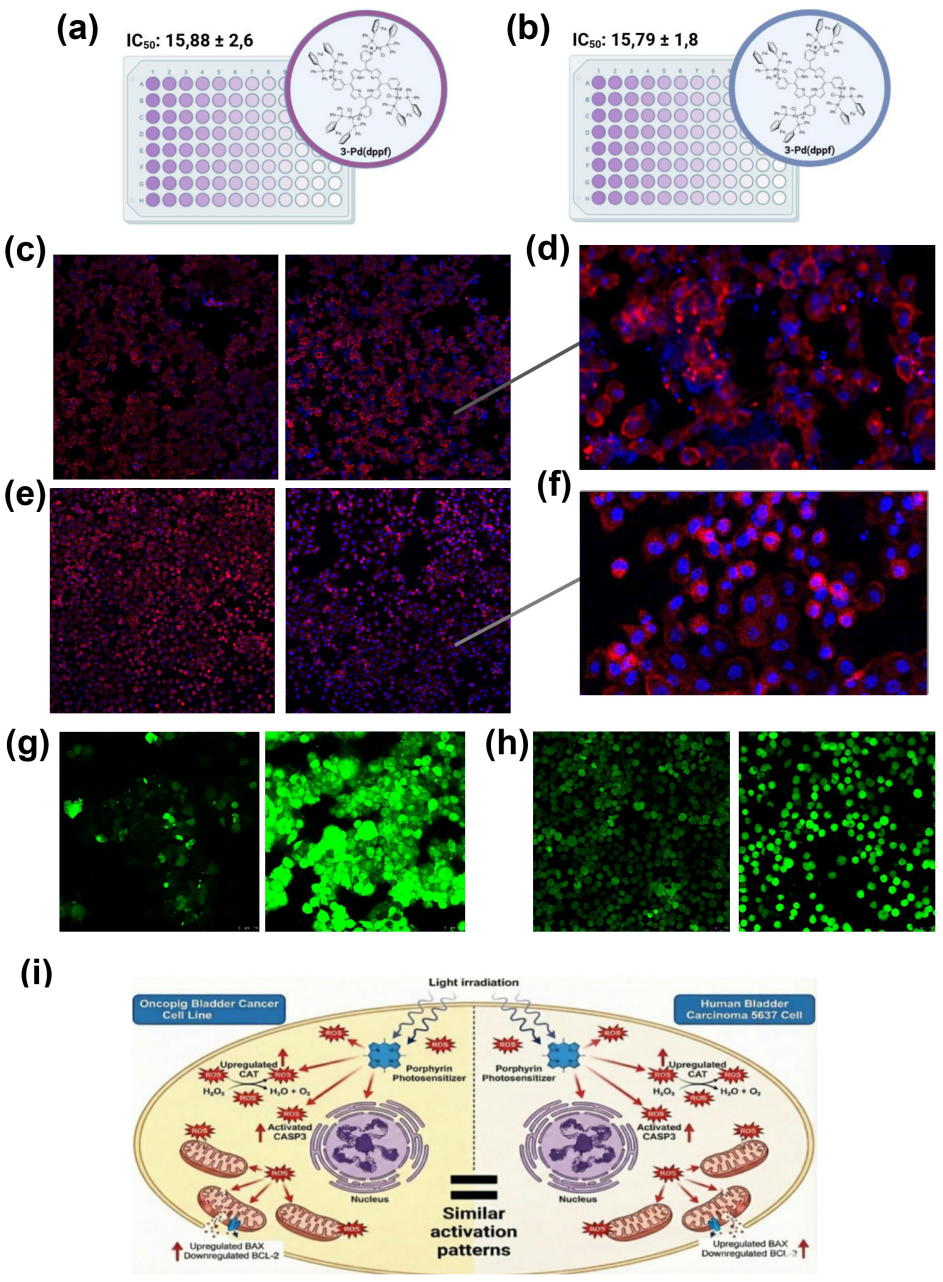
Integrated photodynamic response induced by 3-Pd(dppf) in Oncopig-derived and 5637 bladder cancer models. **(A, B)** IC_50_ determination in Oncopig-derived bladder cancer cells and 5637 human bladder cancer cells, respectively, after treatment with 3-Pd(dppf) under light exposure. **(C–F)** Representative fluorescence microscopy images of DAPI and Texas Red staining in Oncopig-derived and 5637 cells under light control conditions and after treatment with 3-Pd(dppf) followed by light activation. DAPI staining highlights nuclear morphology, while Texas Red staining indicates cytoplasmic/cytoskeletal organization. Higher-magnification images highlight treatment-associated nuclear condensation, cytoplasmic disruption, and loss of cellular organization. **(G, H)** Intracellular ROS generation assessed by DCFH-DA fluorescence in Oncopig-derived and 5637 cells under light control conditions and after 3-Pd(dppf)-mediated photodynamic treatment. Increased green fluorescence indicates ROS accumulation. Quantitative fluorescence data are presented as mean ± SD. **(I)** Schematic representation of the shared photodynamic response observed in Oncopig-derived and 5637 bladder cancer cells, integrating ROS generation, oxidative stress response, and apoptosis-associated molecular changes. RT-qPCR data included in the schematic interpretation are presented as mean ± SEM. All quantitative data were obtained from three independent biological replicates.

Finally, **Figure 2** integrates the key photodynamic events observed in Oncopig-derived and 5637 bladder cancer cells treated with 3-Pd(dppf). The comparable IC_50_ values, ROS accumulation, nuclear condensation, cytoplasmic/cytoskeletal alterations, and apoptosis-associated gene expression changes support a shared response profile between the Oncopig-derived model and human bladder cancer cells. In this integrated analysis, IC_50_, ROS, and DAPI/Texas Red-derived quantitative data are presented as mean ± SD, while RT-qPCR data are presented as mean ± SEM. These findings reinforce the relevance of Oncopig-derived *in vitro* models as a preclinical screening platform for identifying photosensitizers with sufficient biological activity to justify future large-animal validation.

## 4 Discussion

The present study establishes a preclinical *in vitro* screening framework to evaluate palladium(II)-porphyrin photosensitizers for bladder cancer photodynamic therapy using human and Oncopig-derived bladder cancer cell lines. The MTT and LIVE/DEAD assays demonstrated a clear light-dependent cytotoxic effect for the three Pd(II)-complexed meso-tetra-cationic porphyrins tested, with IC_50_ values in the nanomolar range under illumination and minimal or absent toxicity under dark conditions. This photoselective profile is a defining characteristic of effective PDT agents, since cytotoxicity should be preferentially triggered only after light activation of the photosensitizer (23). Importantly, 3-Pd(PEPSI) exhibited higher IC_50_ values than 3-Pd(PPh_3_) and 3-Pd(dppf), indicating lower photodynamic potency. This result supports the value of the proposed screening strategy, as it allowed the early identification of less promising compounds and the prioritization of 3-Pd(PPh_3_) and 3-Pd(dppf) for subsequent mechanistic analyses.

The differences observed among the three compounds suggest that ligand coordination and metal-ligand interactions can strongly influence photodynamic efficacy, in agreement with previous studies using metalated porphyrins in cancer models (21, 24). The three palladium(II)-porphyrins evaluated in this study were previously characterized and shown to possess photophysical properties compatible with photodynamic applications, including visible-light absorption, singlet oxygen generation, low aggregation under biologically relevant conditions, and good photostability under white-light irradiation (20). Importantly, all three compounds share the same principal absorption maximum (Soret band) at 418 nm (20). Because the white LED used in the present study emitted across the 400–800 nm range, this absorption band was encompassed within the irradiation spectrum, allowing comparative photodynamic screening under identical illumination conditions. Although all three compounds share the same palladium(II)-porphyrin core, 3-Pd(PEPSI) consistently exhibited higher IC_50_ values than 3-Pd(PPh_3_) and 3-Pd(dppf) across all bladder cancer cell lines evaluated. Since the porphyrinic macrocycle, the principal absorption band, and the irradiation conditions were identical among the compounds, the observed differences are likely associated with the distinct ligands coordinated to the palladium center. Variations in ligand structure may influence photophysical behavior, molecular interactions, cellular uptake, intracellular distribution, and ultimately photodynamic performance. In contrast, the lower photodynamic potency of 3-Pd(PEPSI) may reflect ligand-dependent differences affecting one or more of these parameters. However, because intracellular uptake, subcellular localization, singlet oxygen production in biological systems, and other mechanistic parameters were not evaluated in the present study, these possibilities should be regarded as hypotheses requiring further investigation rather than demonstrated mechanisms.

The activity of 3-Pd(PPh_3_) and 3-Pd(dppf) also aligns with prior data from our group using related palladium(II) meso-tetra(pyridyl) porphyrin derivatives in human and canine melanoma cell lines, reinforcing the photodynamic potential of this chemical framework across tumor types (20). However, the present study expands this concept by applying these compounds to bladder cancer models and by incorporating an Oncopig-derived *in vitro* system as a translationally relevant intermediate step before future large-animal validation.

The dose-response behavior observed in the MTT assay further supports the photodynamic nature of the cytotoxic effect, with progressive reductions in metabolic activity after light exposure. The enhanced phototoxicity of palladium-containing porphyrins may be partly explained by the heavy-atom effect, which favors intersystem crossing and increases triplet-state yields, thereby enhancing the generation of reactive oxygen species, including singlet oxygen (25, 26). Consistent with this mechanism, DCFH-DA fluorescence microscopy demonstrated increased intracellular ROS generation in all human and Oncopig-derived bladder cancer models treated with 3-Pd(PPh_3_) or 3-Pd(dppf) under light exposure, whereas dark-treated cells exhibited only basal fluorescence. Although DCFH-DA is widely used as an indicator of intracellular oxidative activity, it is an indirect and non-specific probe that does not discriminate among individual ROS species and therefore cannot be interpreted as a direct measurement of singlet oxygen. Nevertheless, these findings confirm that photoactivation is required for oxidative activity and support an oxidative stress-mediated mechanism of cytotoxicity.

Although the magnitude and pattern of CAT, SOD, and GPx regulation varied among cell lines and compounds, the overall response was consistent with cellular adaptation to photoinduced oxidative stress. Catalase plays an important antioxidant role by converting hydrogen peroxide into water and molecular oxygen, thereby contributing to cellular defense against oxidative damage (27). In this study, CAT upregulation was observed in several light-treated groups, particularly after 3-Pd(PPh_3_) exposure, suggesting activation of antioxidant defense mechanisms in response to ROS accumulation. Nevertheless, the simultaneous increase in ROS fluorescence and reduction in cell viability indicates that the oxidative burden generated by PDT was sufficient to overcome these protective responses and trigger cell death.

Downstream of ROS generation, the modulation of apoptosis-associated markers indicates that photodynamic cytotoxicity was associated with activation of cell death pathways (28). In Oncopig-derived cells, 3-Pd(PPh_3_) increased CASP3, CASP8, CASP9, and the BAX/BCL-2 ratio under light exposure, while 3-Pd(dppf) increased CASP3 and the BAX/BCL-2 ratio. These findings suggest activation of apoptosis-associated signaling after photoinduced oxidative stress. In human bladder cancer cells, the apoptotic response was also evident, although the pattern of caspase modulation varied among RT4, 5637, and T24 cells. This variability is expected in cancer models with distinct biological backgrounds and reinforces the importance of using multiple cell lines during preclinical screening rather than relying on a single model (29, 30).

The fluorescence-based morphological assays provided complementary evidence supporting the gene expression findings. DAPI staining revealed increased nuclear condensation and fragmentation in light-treated cells exposed to 3-Pd(PPh_3_) or 3-Pd(dppf), while Texas Red staining showed cytoplasmic and cytoskeletal alterations compatible with loss of cellular integrity. These morphological changes were not evident under dark conditions, further confirming the light-dependent nature of the observed effects. The observed nuclear alterations, including chromatin condensation and nuclear fragmentation, are commonly associated with apoptotic cell death. Although these features were not quantified using dedicated morphometric analyses, they were consistently observed in light-treated groups and interpreted in conjunction with the LIVE/DEAD, ROS, and RT-qPCR results. Together, the cytotoxicity, ROS, gene expression, and imaging data indicate that 3-Pd(PPh_3_) and 3-Pd(dppf) induce photodynamic cell death through an oxidative stress-mediated process associated with apoptosis-related molecular and morphological changes.

A central aspect of this study is the use of Oncopig-derived bladder cancer cells as part of a translational *in vitro* screening pipeline. Previous characterization of this model demonstrated that Oncopig bladder cancer cells recapitulate relevant features of human bladder cancer and reproduce treatment responses *in vitro* (31). In the present study, Oncopig-derived cells exhibited light-dependent cytotoxicity, ROS accumulation, oxidative stress responses, apoptosis-associated gene modulation, and nuclear/cytoplasmic alterations that were broadly comparable to those observed in human bladder cancer cell lines. The integrated analysis shown in **Figure 2**, particularly comparing Oncopig-derived and 5637 cells treated with 3-Pd(dppf), supports the biological convergence between the porcine-derived model and human bladder cancer cells. This does not imply that the Oncopig-derived *in vitro* model fully replaces human models or *in vivo* validation, but it supports its use as a relevant screening layer within a stepwise translational development strategy.

This point is particularly important from the perspective of the 3Rs. The use of Oncopig-derived *in vitro* models allows early exploratory testing of photosensitizers before advancing to animal studies, contributing to Replacement, Reduction, and Refinement in preclinical research (12, 13). In the present study, this approach enabled the exclusion of 3-Pd(PEPSI) from subsequent mechanistic assays due to its lower photodynamic potency, while prioritizing 3-Pd(PPh_3_) and 3-Pd(dppf) based on their stronger light-dependent cytotoxicity, minimal dark toxicity, ROS generation, and apoptosis-associated responses. Therefore, the proposed platform may reduce the number of compounds requiring future *in vivo* testing and refine subsequent experimental designs by informing concentration ranges, light exposure parameters, biological endpoints, and safety considerations before large-animal studies are initiated.

Within the context of bladder cancer PDT, this screening strategy is especially relevant because the bladder is anatomically accessible to localized light delivery and image-guided therapeutic approaches (5, 16). Clinically used photosensitizers for PDT, including Photofrin^®^ (porfimer sodium), have demonstrated therapeutic benefit in bladder cancer, particularly in patients with carcinoma *in situ* and recurrent non-muscle-invasive disease. However, their clinical application may be limited by prolonged skin photosensitivity, limited tissue penetration, suboptimal tumor selectivity and variable treatment responses (5, 32). Consequently, substantial efforts have been directed toward the development of newer photosensitizers with improved photophysical properties, enhanced tumor selectivity and reduced adverse effects (33). In the present study, the palladium(II)-porphyrin derivatives exhibited light-dependent cytotoxicity in the nanomolar range together with minimal dark toxicity, indicating promising photodynamic activity. However, because no clinically approved photosensitizer was evaluated under identical experimental conditions, direct comparisons of efficacy cannot be made, and controlled head-to-head studies will be required to determine their relative therapeutic performance. Importantly, the present study should not be interpreted as a direct validation of intravesical PDT. Rather, it provides an *in vitro* preclinical foundation for selecting photosensitizers with sufficient biological activity and mechanistic plausibility to justify future evaluation in more complex systems. In this sense, Oncopig-derived *in vitro* models represent an intermediate translational step between conventional human cancer cell assays and future Oncopig-based *in vivo* studies requiring clinically relevant bladder anatomy, controlled illumination geometry, photosensitizer exposure, and treatment monitoring.

This study has limitations that should be acknowledged. First, the experiments were performed in two-dimensional cell culture systems and therefore do not reproduce the full complexity of tumor architecture, urothelial barrier function, oxygen gradients, immune interactions, or intravesical pharmacokinetics. Second, although the compounds showed strong light-dependent cytotoxicity and low dark toxicity, active molecular targeting, selective uptake mechanisms, subcellular localization, and comparative toxicity against non-malignant urothelial cells were not addressed. Third, the use of broad-spectrum white light provides functional evidence of photodynamic activation, but future studies should optimize wavelength-specific excitation, fluence, treatment geometry, and cystoscopy-compatible illumination conditions. These limitations define the next translational steps and reinforce the need for a staged development pipeline that begins with *in vitro* screening and progresses only selected candidates to ethically justified large-animal validation.

Overall, the results demonstrate that 3-Pd(PPh_3_) and 3-Pd(dppf) are the most promising photosensitizers among the compounds tested, showing nanomolar phototoxicity, minimal dark toxicity, ROS-mediated activity, and apoptosis-associated responses in both human and Oncopig-derived bladder cancer models. The concordance between Oncopig-derived and human cellular responses supports the use of this platform as a 3R-oriented preclinical screening approach for bladder cancer PDT. By identifying biologically active photosensitizers before *in vivo* testing, this strategy contributes to a more rational, ethical, and translationally grounded pathway for the future development of localized and image-guided PDT protocols.

## 5 Conclusion

This study demonstrates that preclinical *in vitro* screening using human and Oncopig-derived bladder cancer models is a valuable strategy for identifying palladium(II)-porphyrin photosensitizers with translational potential for bladder cancer photodynamic therapy. Among the three compounds evaluated, 3-Pd(PPh_3_) and 3-Pd(dppf) showed the most favorable photodynamic profiles, combining nanomolar light-dependent cytotoxicity, minimal dark toxicity, intracellular ROS generation, modulation of oxidative stress responses, and apoptosis-associated molecular and morphological changes. In contrast, 3-Pd(PEPSI) displayed lower phototoxic potency, supporting its exclusion from subsequent mechanistic analyses and illustrating the practical value of an initial screening step.

Importantly, the Oncopig-derived bladder cancer model exhibited photodynamic responses broadly comparable to those observed in human bladder cancer cell lines, supporting its use as a biologically relevant *in vitro* platform within a stepwise translational pipeline. This approach does not replace the need for future validation in more complex systems, but it provides a rational and ethically responsible layer for prioritizing photosensitizer candidates before progression to large-animal studies.

Overall, our findings position 3-Pd(PPh_3_) and 3-Pd(dppf) as promising photosensitizers for further bladder cancer PDT development and support the use of Oncopig-derived *in vitro* models as a 3R-oriented preclinical screening platform. By enabling early candidate selection, mechanistic characterization, and refinement of experimental parameters, this strategy contributes to a more rational, responsible, and translationally grounded pathway for the future development of localized and image-guided PDT approaches.

## Data Availability

The original contributions presented in the study are included in the article/**Supplementary Material**. Further inquiries can be directed to the corresponding author.
