# Supplementary material for: Preclinical translational screening of palladium(II)-porphyrin photosensitizers across human and Oncopig bladder cancer cell lines
Source: Front Oncol. 2026 Jul 9;16:1882402. doi: 10.3389/fonc.2026.1882402 (PMC13391413; doi:10.3389/fonc.2026.1882402)
Supplement: Supplementary file 1 [file DataSheet1.pdf]

Supplementary information

Table S1. Primer sequences used for RT-qPCR analysis. The table presents the forward (F) and reverse (R) primer sequences (5′–3′). The genes analyzed include GAPDH (Glyceraldehyde-3-phosphate dehydrogenase), SOD (Superoxide dismutase), CAT (Catalase), GPx (Glutathione peroxidase), BAX **(**BCL2-associated X protein), BCL2 (B-cell lymphoma 2), Casp 8 (Caspase 8), Casp 9 (Caspase 9), and Casp 3 (Caspase 3). Primer sets were designed for both human and porcine genes.

Species	Primer	Sequence
Human	**GAPDH**	F: ACAACTTTGGTATCGTGGAAGG<br>R: GCCATCACGCCACAGTTTC
Pig	**GAPDH**	F: ACTCACATCTTCTACCTTTGATGCT<br>R: TGTTGCTGTAGCCAAATTCA
Human	**SOD**	F: AGGGCATCATCAATTTCGAG<br>R: TGCCTCTCTTCATCCTTTGG
Pig	**SOD**	F: ACTCACCTCTCTTGATCCTTTGGCCCACC<br>R: TCCTCACTTCAATCCTGAATCCAA
Human	**CAT**	F: TTTCCCAGGAAGATCCTGAC<br>R: ACCTTGGTGAGATCGAATGG
Pig	**CAT**	F: CTTGGAACATTGTACCCGCT<br>R: GTCCAGAAGAGCCTGAATGC
Human	**GPx**	F: TTCCCGTGCAACCAGTTTG<br>R: TTCACCTCGCACTTCTCGAA
Pig	**GPx**	F: CAAGAATGGGGAGATCCTGA<br>R: GATAAACTTGGGGTCGGTCA
Human	**BAX**	F: ATGCGTCCACCAAGAAGC<br>R: ACGGCGGCAATCATCCTC
Pig	**BAX**	F: TGCCTCAGGATGCATCTACC<br>R: AAGTAGAAAAGCGCGACCAC
Human	**BCL2**	F: GGTGGGGTCATGTGTGTGG<br>R:CGGTTCAGGTACTCAGTCATCC
Pig	**BCL2**	F: AGGGCATTCAGTGACCTGAC<br>R: CGATCCGACTCACCAATACC
Human	**Casp 8**	F: GGATGGCCACTGTGAATAACTG<br>R: TCGAGGACATCGCTCTCTCA
Pig	**Casp 8**	F: AGACAAGGGCATCATCATCGG<br>R: GGTTTACCAAGAAGGGAACGG
Human	**Casp 9**	F: CCAGAGATTCGCAAACCAGAGG<br>R: GAGCACCGACATCACCAAATCC
Pig	**Casp 9**	F: AATGCCGATTTGGCTTACGT<br>R: CATTTGCTTGGCAGTCAGGTT
Human	**Casp 3**	F: CAGTGGAGGCCGACTTCTTG<br>R: TGGCACAAAGCGACTGGAT
Pig	**Casp 3**	F:CGTGCTTCTAAGCCATGGTG<br>R: GTCCCACTGTCCGTCTCAAT

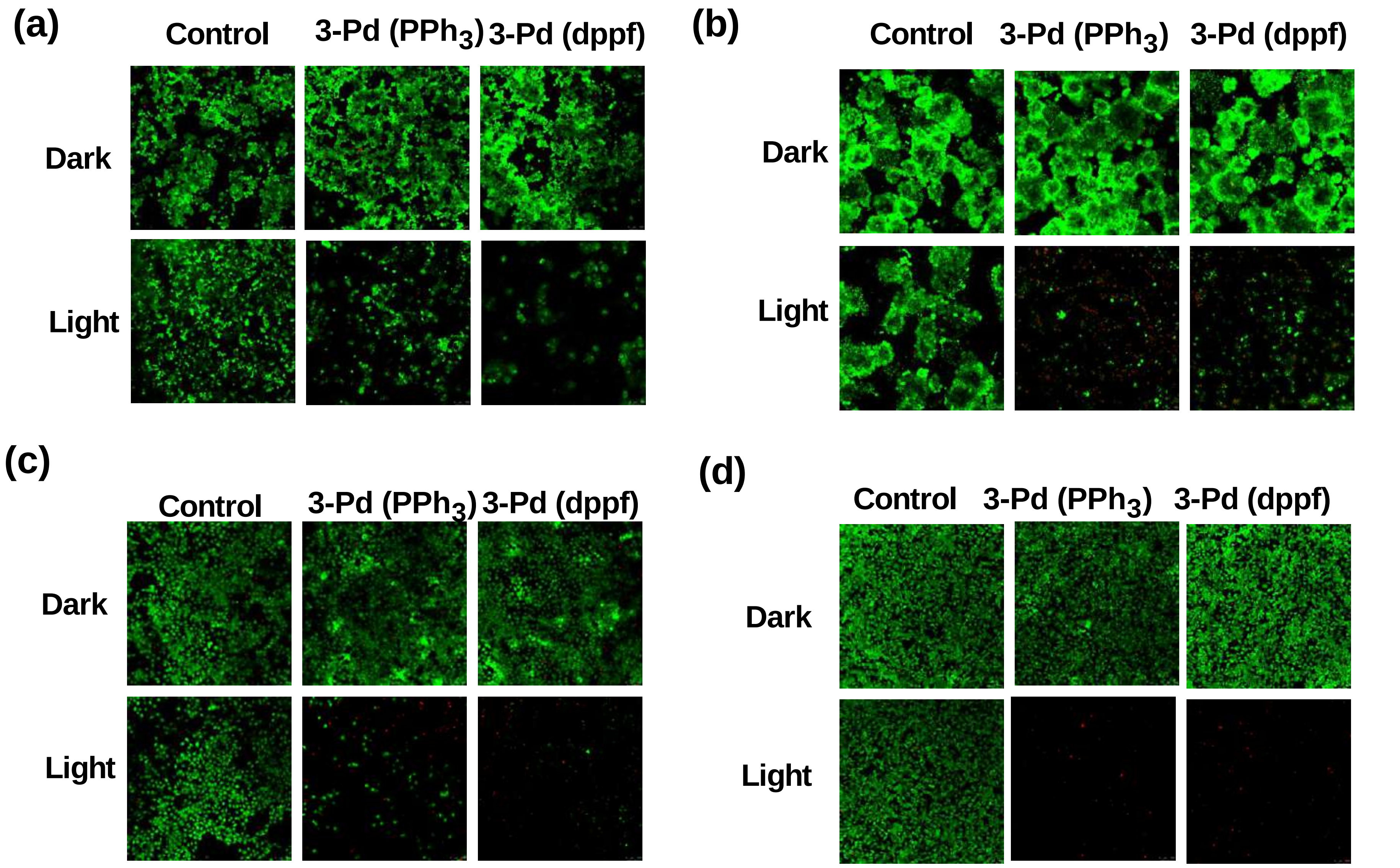

Figure S1. LIVE/DEAD Cell Viability Assay after treatment with palladium-based porphyrin photosensitizers under light and dark conditions. Confocal microscopy images of **(a)** Oncopig, **(b)** RT4, **(c)** 5637, and **(d)** T24 cell lines treated with the porphyrins 3-Pd(PPh_3) and 3-Pd(dppf), acquired 24 h after photodynamic therapy (Light) or in the absence of irradiation (Dark). Calcein-AM staining (green, emission ~515 nm) indicates viable cells, while EthD-1 staining (red, emission ~635 nm) indicates dead cells. Scale bars: 100 µm. Images shown are representative of three independent biological replicates.

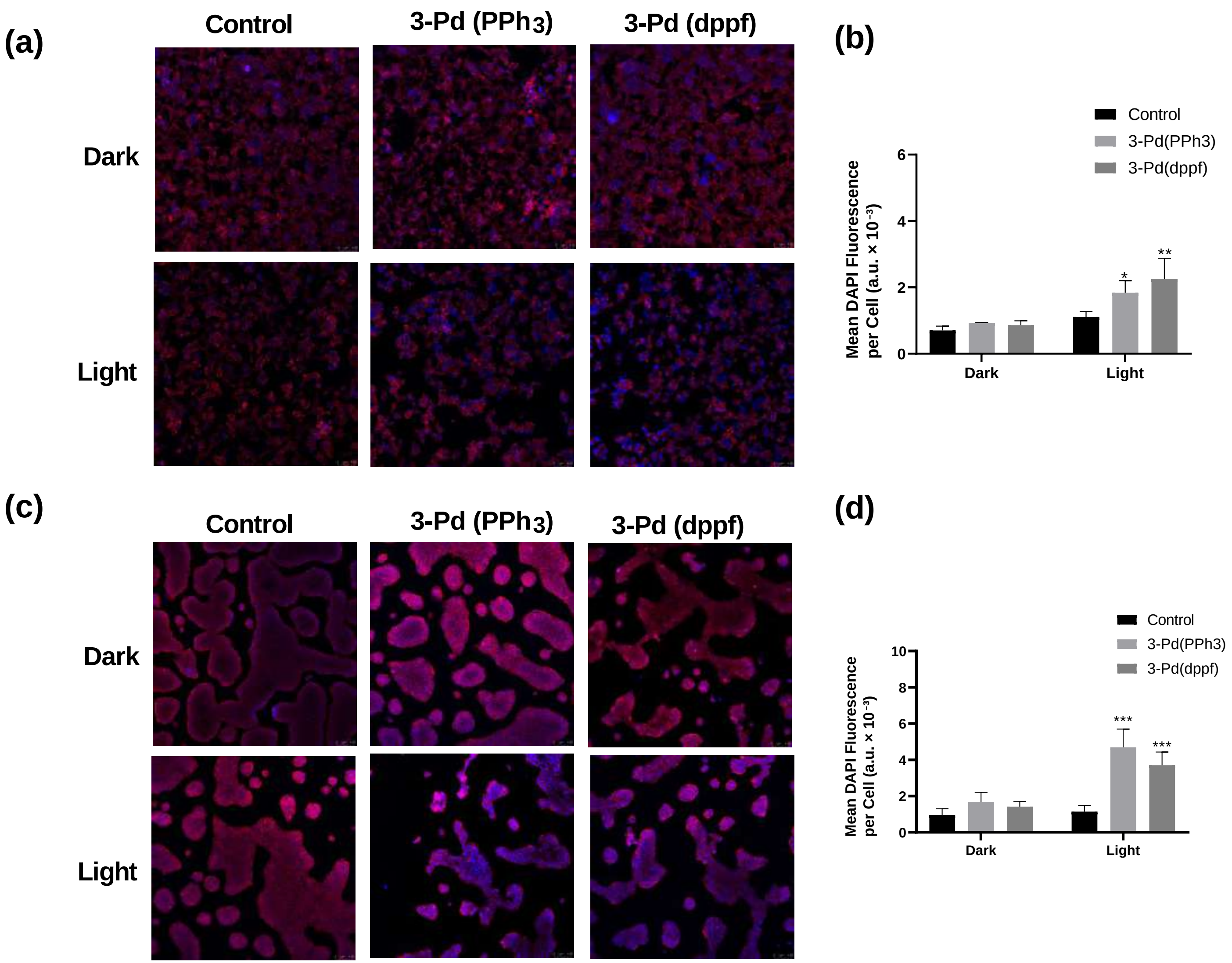

Figure S2. Fluorescence imaging of nuclei (DAPI) and cytoplasmic staining (Texas Red) in bladder cancer cell lines following treatment with palladium-based porphyrin photosensitizers under light and dark conditions. **(a)** Oncopig cell line. Fluorescence microscopy images of cells treated with 3-Pd(PPh_3) and 3-Pd(dppf), acquired 24 h after photodynamic therapy (Light) or under non-irradiated conditions (Dark). DAPI staining (blue, emission ~461 nm) marks cell nuclei, while Texas Red staining (red, emission ~615 nm) labels cytoplasmic content. Scale bars: 100 µm. **(b)** Oncopig cell line. Quantification of mean DAPI fluorescence per cell obtained from three independent fields of view for each condition. Results are expressed as mean ± SD. Statistical significance was determined by two-way ANOVA. *$p < 0.05$; **$p < 0.01$; ***$p < 0.001$. **(c)** RT4 cell line. Fluorescence microscopy images of cells treated with 3-Pd(PPh_3) and 3-Pd(dppf) under Light and Dark conditions, acquired 24 h after treatment. DAPI (blue) highlights nuclei and Texas Red (red) highlights the cytoplasmic region. Scale bars: 100 µm. **(d)** RT4 cell line. Quantification was performed using three distinct fields of view per sample from three independent biological replicates. Results are expressed as mean ± SD. Statistical significance was determined by two-way ANOVA. *$p < 0.05$; **$p < 0.01$; ***$p < 0.001$.

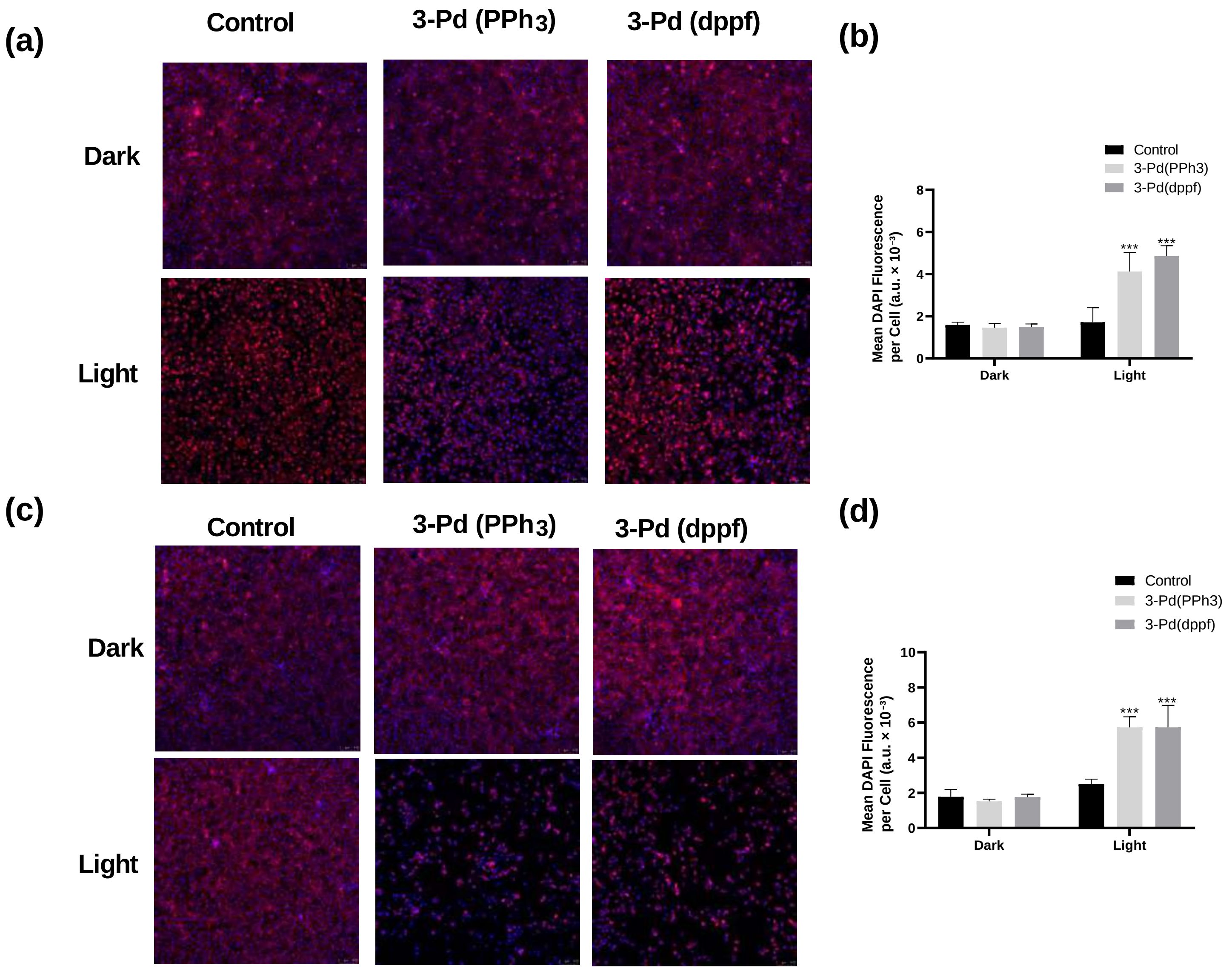

Figure S3. Fluorescence imaging of nuclei (DAPI) and cytoplasmic staining (Texas Red) in bladder cancer cell lines following treatment with palladium-based porphyrin photosensitizers under light and dark conditions. **(a)** 5637 cell line. Fluorescence microscopy images of cells treated with 3-Pd(PPh_3) and **3**-Pd(dppf), acquired 24 h after photodynamic therapy (Light) or under non-irradiated conditions (Dark). DAPI staining (blue, emission ~461 nm) marks cell nuclei, while Texas Red staining (red, emission ~615 nm) labels cytoplasmic content. Scale bars: 100 µm. **(b)** 5637 cell line. Quantification of mean DAPI fluorescence per cell obtained from three independent fields of view for each condition. Results are expressed as mean ± SD. Statistical significance was determined by two-way ANOVA. *$p < 0.05$; **$p < 0.01$; ***$p < 0.001$. **(c)** T24 cell line. Fluorescence microscopy images of cells treated with 3-Pd(PPh_3) and 3-Pd(dppf) under Light and Dark conditions, acquired 24 h after treatment. DAPI (blue) highlights nuclei and Texas Red (red) highlights the cytoplasmic region. Scale bars: 100 µm **.(d)** T24 cell line Quantification was performed using three distinct fields of view per sample from three independent biological replicates. Results are expressed as mean ± SD. Statistical significance was determined by two-way ANOVA. *$p < 0.05$; **$p < 0.01$; ***$p < 0.001$.

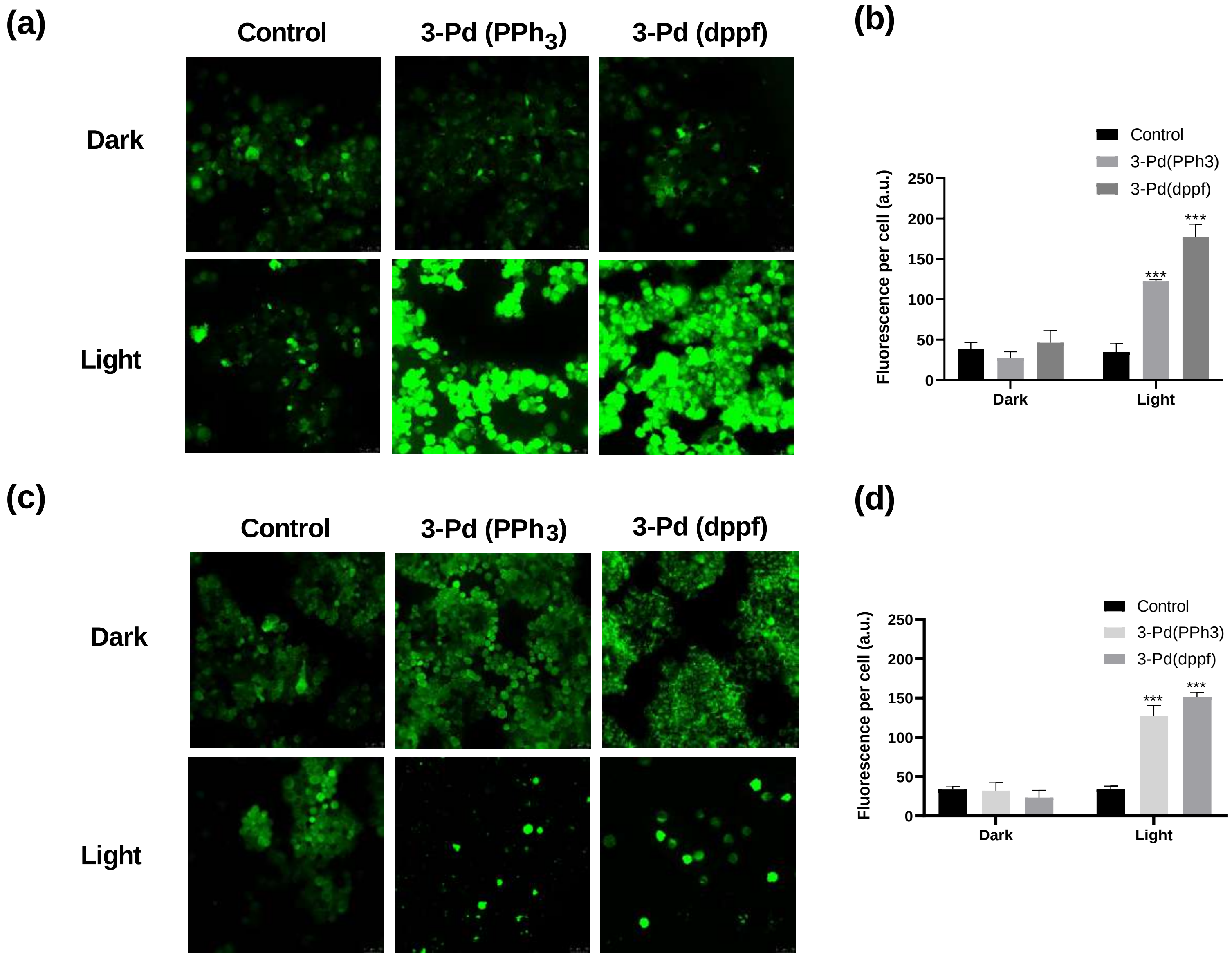

Figure S4. Intracellular reactive oxygen species (ROS) generation after treatment with palladium-based porphyrin photosensitizers under light and dark conditions. **(a)** Oncopig cell line. Confocal microscopy images of cells treated with 3-Pd(PPh_3) and 3-Pd(dppf)at their respective IC_{50} concentrations, acquired 24 h after photodynamic therapy (Light) or in the absence of irradiation (Dark). Intracellular ROS production was detected using the DCFH-DA probe; green fluorescence (DCF, emission ~525 nm) indicates ROS generation. Scale bars: 100 µm. **(b)** Oncopig cell line. Quantification of intracellular ROS levels expressed as fluorescence per cell, obtained from three distinct regions per well. Results are expressed as mean ± SD. Statistical significance was determined by two-way ANOVA. $^*p < 0.05$; $^{**}p < 0.01$; $^{***}p < 0.001$. **(c)** RT4 cell line. Confocal microscopy images of cells treated with 3-Pd(PPh_3) and 3-Pd(dppf), imaged 24 h after PDT exposure (Light) or under dark conditions (Dark). Green fluorescence corresponds to DCF formation and reflects intracellular ROS production. Scale bars: 100 µm. **(d)** RT4 cell line. Quantification was performed using three distinct fields of view per sample from three independent biological replicates. Results are expressed as mean ± SD. Statistical significance was determined by two-way ANOVA. $^*p < 0.05$; $^{**}p < 0.01$; $^{***}p < 0.001$.

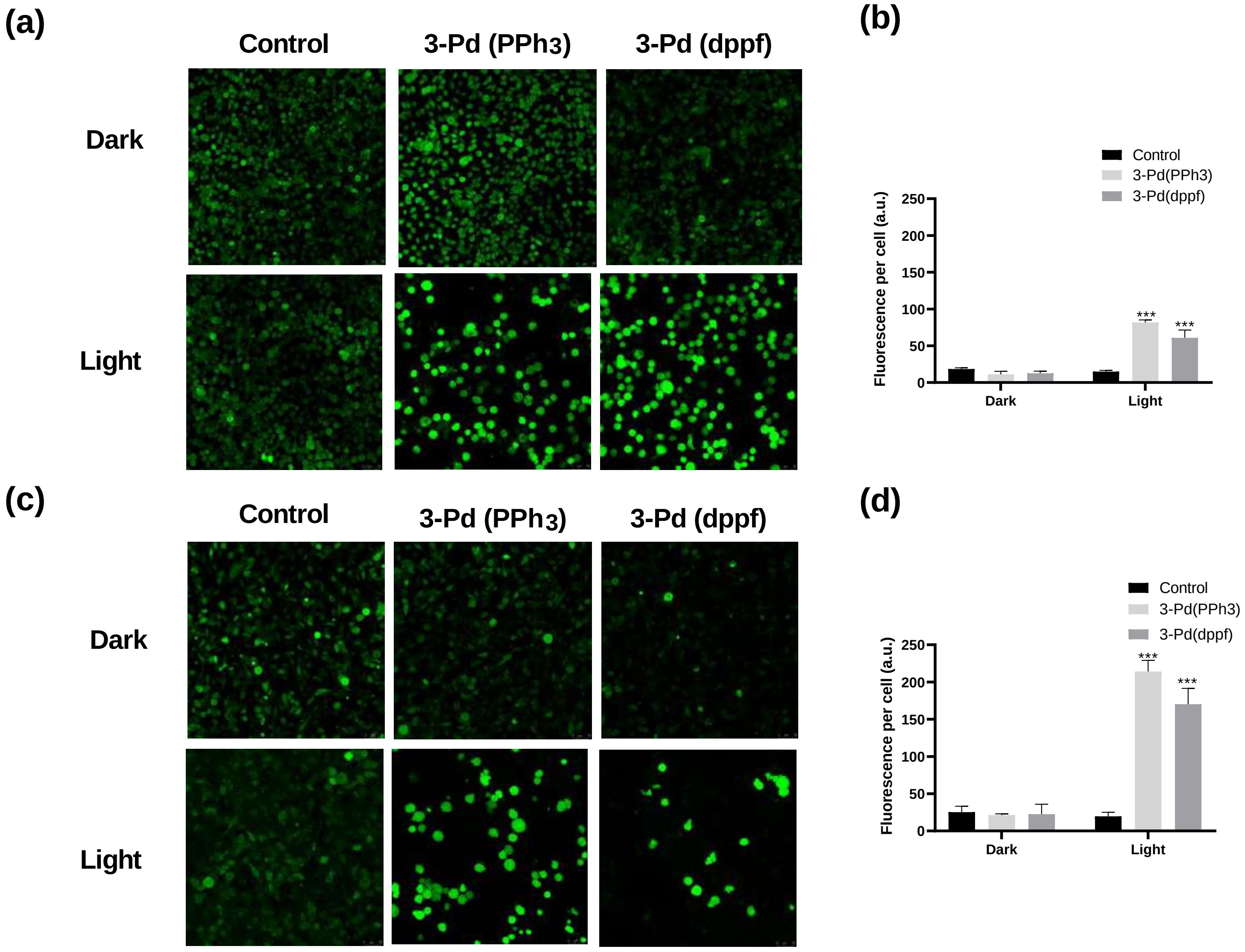

Figure S5. Intracellular reactive oxygen species (ROS) generation after treatment with palladium-based porphyrin photosensitizers under light and dark conditions. **(a)** 5637 cell line. Confocal microscopy images of cells treated with 3-Pd(PPh_3) and 3-Pd(dppf) at their respective IC_{50} concentrations, acquired 24 h after photodynamic therapy (Light) or in the absence of irradiation (Dark). Intracellular ROS production was detected using the DCFH-DA probe; green fluorescence (DCF, emission ~525 nm) indicates ROS generation. Scale bars: 100 µm. **(b)** 5637 cell line. Quantification of intracellular ROS levels expressed as fluorescence per cell, obtained from three distinct regions per well. Results are expressed as mean ± SD. Statistical significance was determined by two-way ANOVA. *$p < 0.05$; **$p < 0.01$; ***$p < 0.001$. **(c)** T24 cell line. Confocal microscopy images of cells treated with 3-Pd(PPh_3) and 3-Pd(dppf) imaged 24 h after PDT exposure (Light) or under dark conditions (Dark). Green fluorescence corresponds to DCF formation and reflects intracellular ROS production. Scale bars: 100 µm**. (d)** T24 cell line. Quantification was performed using three distinct fields of view per sample from three independent biological replicates. Results are expressed as mean ± SD. Statistical significance was determined by two-way ANOVA. *$p < 0.05$; **$p < 0.01$; ***$p < 0.001$.

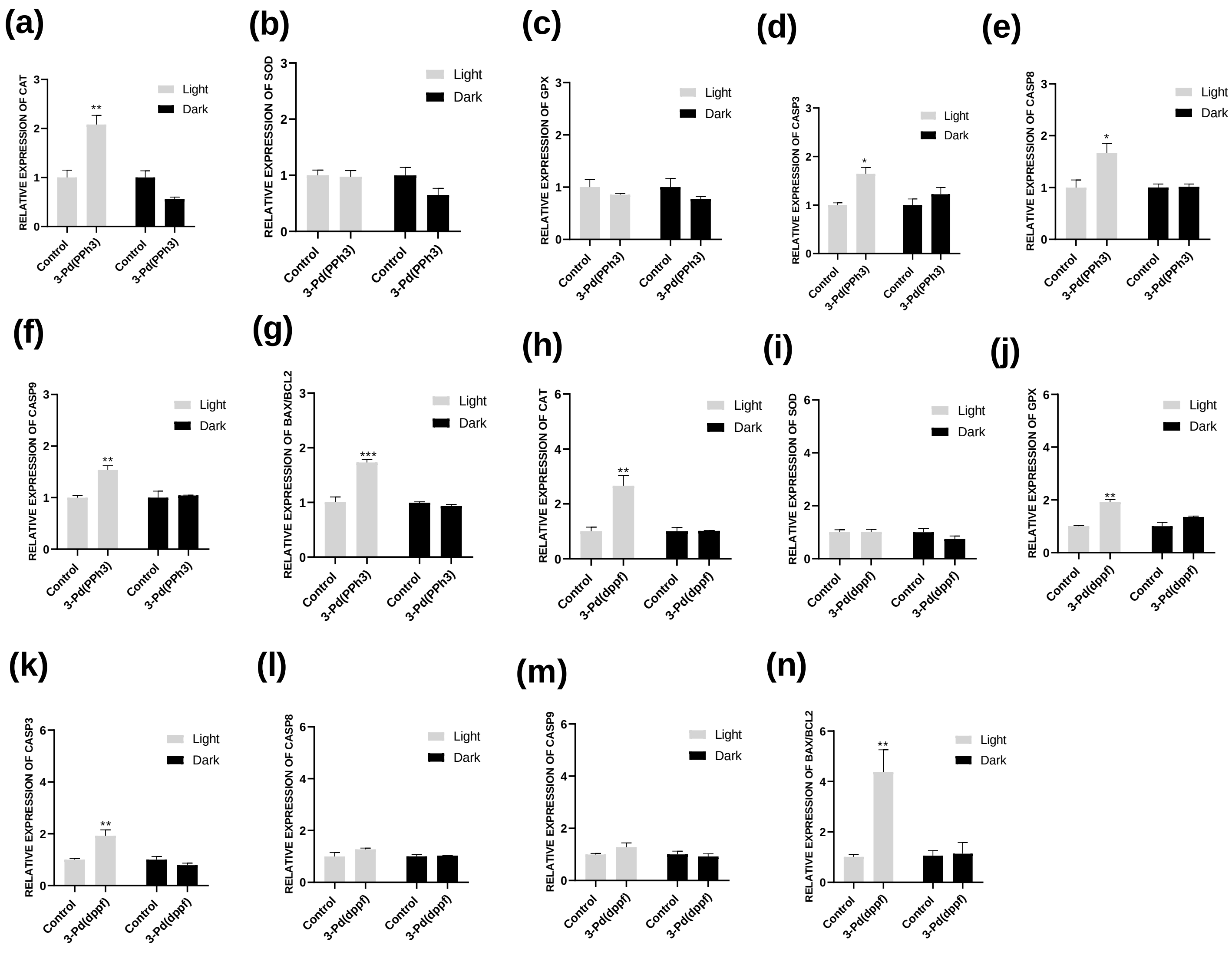

Figure S6. Relative gene expression analysis by RT-qPCR in Oncopig cells treated with the porphyrin complexes 3-Pd(PPh_3) and 3-Pd(dppf) under light and dark conditions. Gene expression levels were normalized to GAPDH and expressed relative to the control group. Antioxidant-related genes CAT, SOD, and GPx are shown in panels **(a–c)** for 3-Pd(PPh_3) and **(h–j)** for 3-Pd(dppf), while apoptosis-related genes CASP3, CASP8, CASP9, and the BAX/BCL-2 ratio are shown in panels **(d–g)** and **(k–n)**, respectively. Data are presented as mean ± SEM from three independent biological replicates. Statistical significance was determined by two-way ANOVA. *$p < 0.05$; **$p < 0.01$; ***$p < 0.001$.

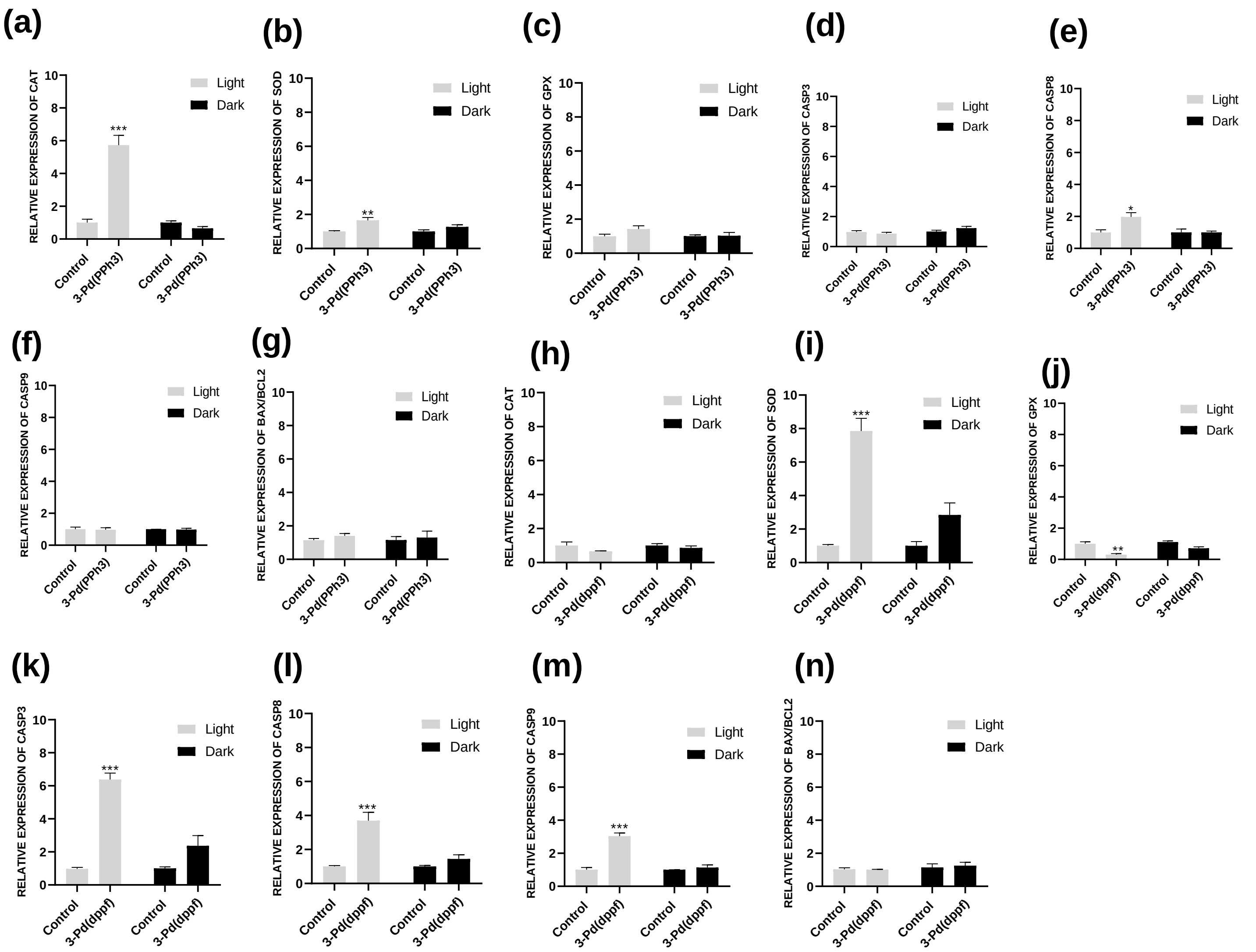

Figure S7. Relative gene expression analysis by RT-qPCR in RT4 bladder cancer cells treated with the porphyrin complexes 3-Pd(PPh_3) and 3-Pd(dppf) under light and dark conditions. Gene expression levels were normalized to GAPDH and expressed relative to the control group. Antioxidant-related genes CAT, SOD, and GPx are shown in panels **(a–c)** for 3-Pd(PPh_3) and **(h–j)** for 3-Pd(dppf), while apoptosis-related genes CASP3, CASP8, CASP9, and the BAX/BCL-2 ratio are shown in panels **(d–g)** and **(k–n)**, respectively. Data are presented as mean ± SEM from three independent biological replicates. Statistical significance was determined by two-way ANOVA. $^*p < 0.05$; $^{**}p < 0.01$; $^{***}p < 0.001$.

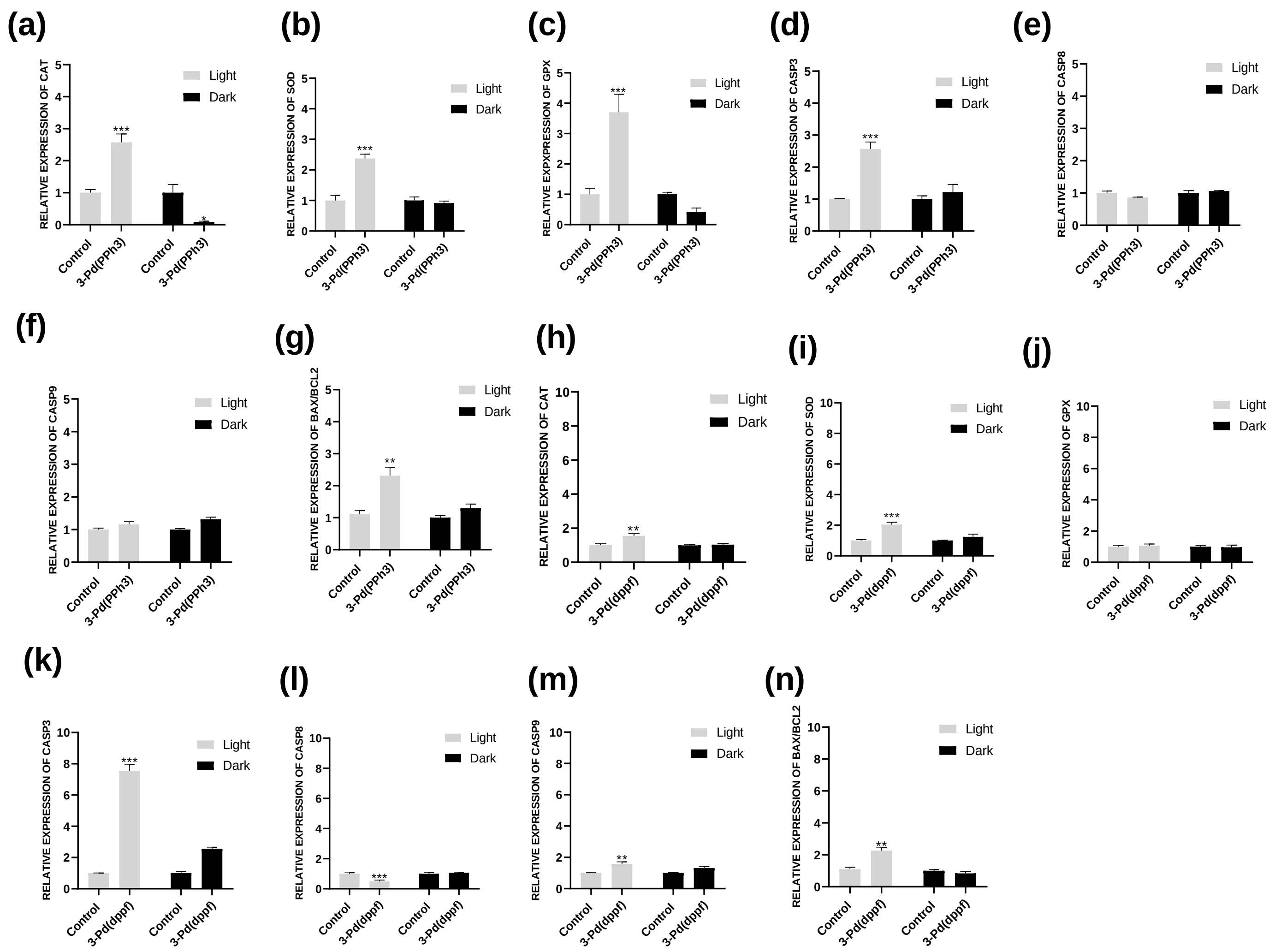

Figure S8. Relative gene expression analysis by RT-qPCR in 5637 human bladder cancer cells treated with the porphyrin complexes 3-Pd(PPh_3) and 3-Pd(dppf) under light and dark conditions. Gene expression levels were normalized to GAPDH and expressed relative to the control group. Antioxidant-related genes CAT, SOD, and GPx are shown in panels **(a–c)** for 3-Pd(PPh_3) and **(h–j)** for 3-Pd(dppf), while apoptosis-related genes CASP3, CASP8, CASP9, and the BAX/BCL-2 ratio are shown in panels **(d–g)** and **(k–n)**, respectively. Data are presented as mean ± SEM from three independent biological replicates. Statistical significance was determined by two-way ANOVA. *$p < 0.05$; **$p < 0.01$; ***$p < 0.001$.

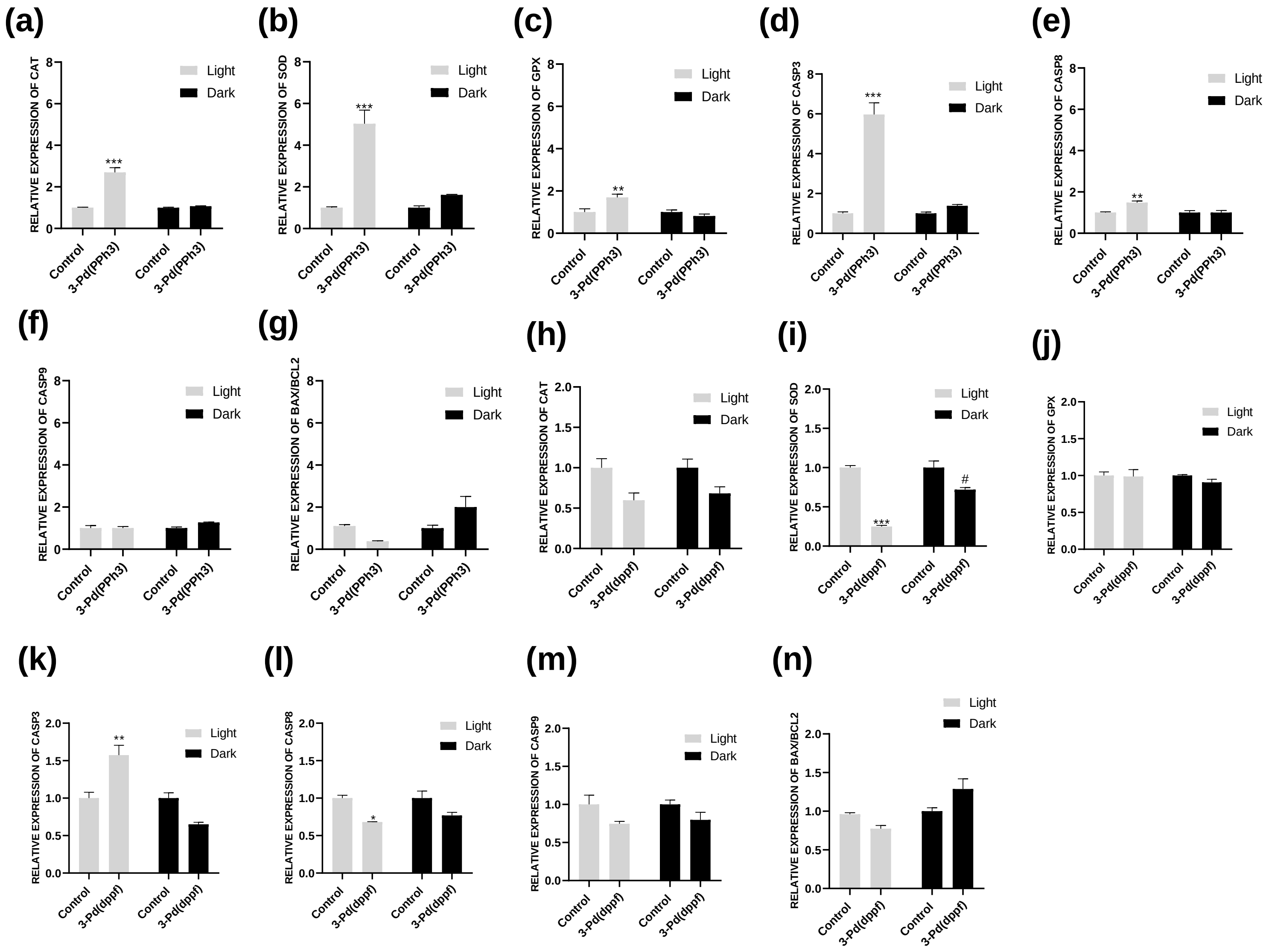

Figure S9. Relative gene expression analysis by RT-qPCR in T24 human bladder cancer cells treated with the porphyrin complexes 3-Pd(PPh_3) and 3-Pd(dppf) under light and dark conditions. Gene expression levels were normalized to GAPDH and expressed relative to the control group. Antioxidant-related genes CAT, SOD, and GPx are shown in panels **(a–c)** for 3-Pd(PPh_3) and **(h–j)** for 3-Pd(dppf), while apoptosis-related genes CASP3, CASP8, CASP9, and the BAX/BCL-2 ratio are shown in panels **(d–g)** and **(k–n)**, respectively. Data are presented as mean ± SEM from three independent biological replicates. Statistical significance was determined by two-way ANOVA. $^*p < 0.05$; $^{**}p < 0.01$; $^{***}p < 0.001$.

Table S2. Ct values of the reference gene GAPDH in all experimental groups and cell lines. Values represent the mean Ct obtained from technical replicates for each biological replicate.

**Groups/Cell lines**	**5637**	**T24**	**RT4**	**Oncopig**
**Control Light**	16,39	15,24	22,61	18,74
	16,63	15,15	22,05	18,53
	16,37	15,52	22,4	18,82
**Control Dark**	16,36	15,31	21,6	19,39
	16,3	15,47	21,87	18,64
	16,15	15,37	21,1	19,015
**3-Pd (PPh3) Light**	16,67	19,3	23,58	18,79
	16,87	19,14	22,68	18,79
	16,4	19,18	22,97	18,79
**3-Pd (PPh3) Dark**	16,62	15,36	22,97	18,79
	16,75	15,25	22,24	18,79
	16,62	15,25	22	18,79
**3-Pd (dppf) Light**	18,5	16,95	24,6	18,99
	19,16	16,15	24,32	19,12
	18,25	16,16	24,46	19,055
**3-Pd (dppf) Dark**	16,75	14,87	23,32	18,81
	16,07	15,09	24,2	18,95
	16,8	14,68	24,68	18,88

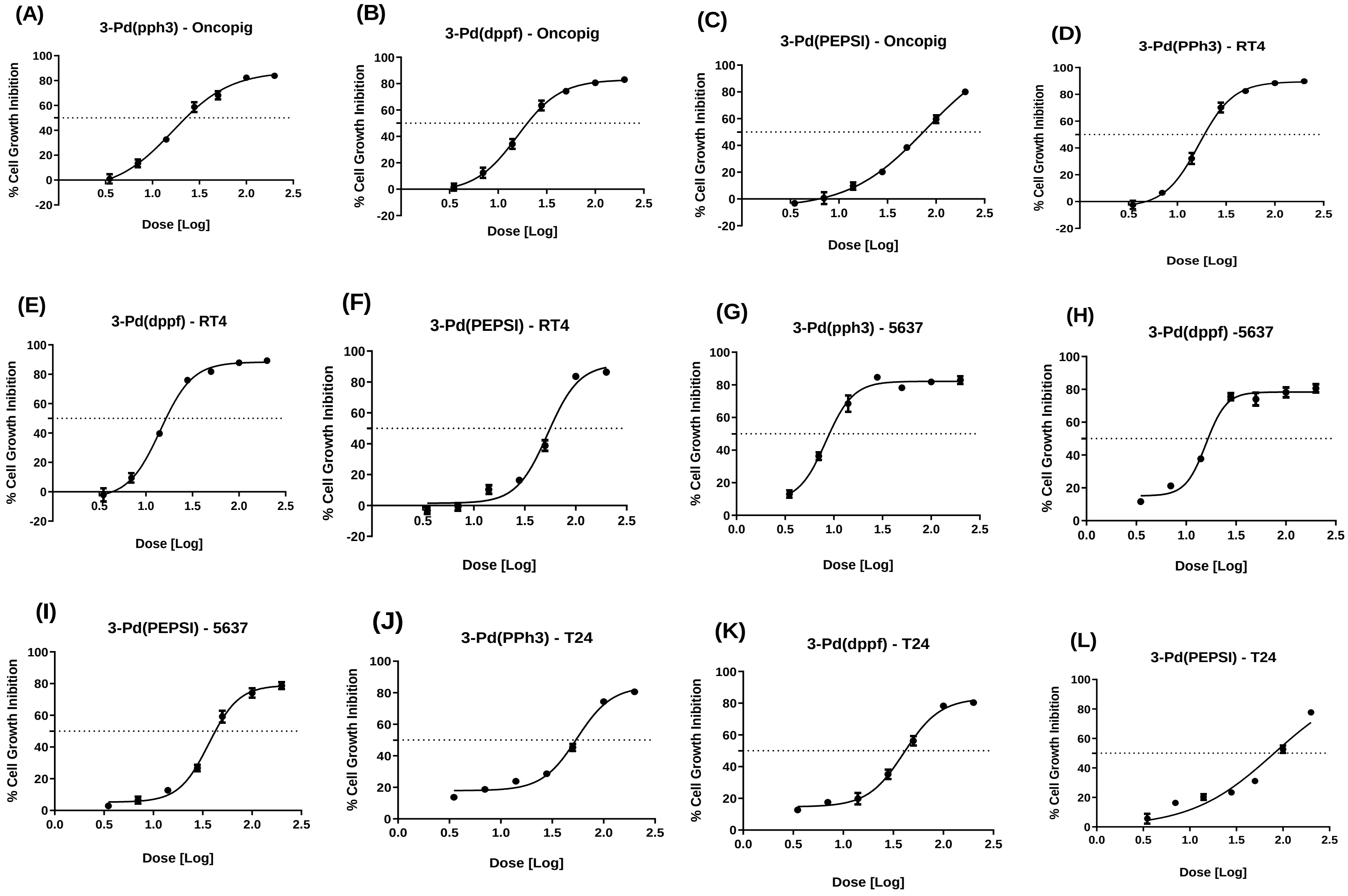

Figure S10. Dose–response curves of palladium(II)-porphyrins in bladder cancer cell lines after photodynamic treatment. Cell growth inhibition (%) was plotted against the logarithm of compound concentration and fitted by nonlinear regression (variable slope model). Panels A–L correspond to the different compounds and cell lines evaluated. The dotted line represents 50% cell growth inhibition (IC_{50} threshold).
